# Little known Orphninae (Coleoptera, Scarabaeidae) collected by the Madagascan expeditions of the California Academy of Sciences

**DOI:** 10.3897/BDJ.1.1016

**Published:** 2013-11-27

**Authors:** Andrey V. Frolov

**Affiliations:** † Zoological Institute of Russian Academy of Sciences, Sankt-Petersburg, Russia

**Keywords:** Scarab beetles, orphnines, new species, *Madecorphnus*, *Triodontus*, Madagascar

## Abstract

New locality records for *Madecorphnus niger* Frolov, 2010, *Madecorphnus falcatus* Paulian, 1992, *Madecorphnus simplex* Frolov, 2010, and *Triodontus itremoi* Paulian, 1977, are given. Endophallus armature of *Madecorphnus niger* Frolov, 2010, is described and illustrated.

## Introduction

Madagascar houses a rich and taxonomically diverse fauna of the scarab beetles of subfamily Orphninae. Thirty five species of four genera are known and all of them are endemic to the island (Frolov 2010, Frolov and Montreuil 2009, Paulian 1977, Paulian 1992). Only three species (*Triodontus nitidulus* (Guérin-Méneville, 1844), *Pseudorphnus coquerelii* (Fairmaire, 1868), and *Renorphnus clementi* (Petrovitz, 1971)) have a reasonably wide distribution and are known from a large number of specimens, which might indicate their adaptation to feeding on crops (documented for *Triodontus nitidulus* larvae: Randriamanantsoa et al. (2010). All other species are rare in collections and many are known from type specimens only. Distribution of taxa is biased towards indigenous forests of the central domain of the Eastern Region and many species ranges may now be limited to isolated patches of forests.

In 2000s, researchers from the California Academy of Sciences (San-Francisco, USA) undertook insect surveys on Madagascar which yielded a rich orphnine material including a few new species (Frolov 2011, Frolov 2012). However, a part of this material, kept in vials with alcohol, was not available for study until recently. In this material, a few rare Madagascan orphnine species were identified. New locality records for these species are given in the present communication. The endophallus armature of *Madecorphnus niger* Frolov, 2010, is described and illustrated.

## Materials and methods

All examined material is housed in the collection of California Academy of Sciences (San-Francisco, USA).

Preparation of genitalia follows the common technique used in entomological research. The photograph was taken with a Leica MZ9.5 stereo microscope and a Leica DFC290 digital camera from specimen in glycerol. Partially focused serial images were combined in Helicon Focus software (Helicon Soft Ltd.) to produce completely focused image. The photograph was not altered except for levels and tone correction in Adobe Photoshop (Adobe Inc.). The distribution map was generated with ArcGIS software. As the base map, a Madagascar vegetation map (CEPF Madagascar Vegetation Mapping Project of Royal Botanic Gardens, Kew, Missouri Botanical Garden, and Conservation International’s Center for Applied Biodiversity Science; http://www.vegmad.org) was used. Co-ordinates of the localities were taken from the specimen labels.

## Taxon treatments

### Madecorphnus niger

Frolov, 2010

#### Materials

- **Type status:** Other material. **Occurrence:** recordedBy: B. Fisher; individualCount: 1; sex: male; **Taxon:** scientificName: Madecorphnus niger; **Location:** country: Madagascar; stateProvince: Antananarivo; verbatimLocality: 3 km 41° NE Andranomay, 11.5 km 147° SSE Anjozorobe; verbatimElevation: 1300 m; verbatimLatitude: 18°28'24"S; verbatimLongitude: 47°57'36"E; **Identification:** identifiedBy: Andrey V. Frolov; **Event:** eventDate: 5-13 Dec 2000; **Record Level:** collectionID: urn:lsid:biocol.org:col:35143; institutionCode: CAS; collectionCode: CASENT
- **Type status:** Other material. **Occurrence:** recordedBy: B. Fisher; individualCount: 1; sex: male; **Taxon:** scientificName: Madecorphnus niger; **Location:** country: Madagascar; verbatimLocality: Toamasina Parc National de Zahamena, Onibe River; verbatimElevation: 780 m; verbatimLatitude: 17°45'33"S; verbatimLongitude: 048°51'17"E; **Identification:** identifiedBy: Andrey V. Frolov; **Event:** eventDate: 21-23 February; **Record Level:** collectionID: urn:lsid:biocol.org:col:35143; institutionCode: CAS; collectionCode: CASENT

#### Distribution

This species was known from 2 types from Perinet (Andasibe). The two additional localities reported here are situated 70 km NNW and 140 km NNE of the type locality, both within the humid forest biome (Fig. 3).

#### Taxon discussion

**Figure 1.**
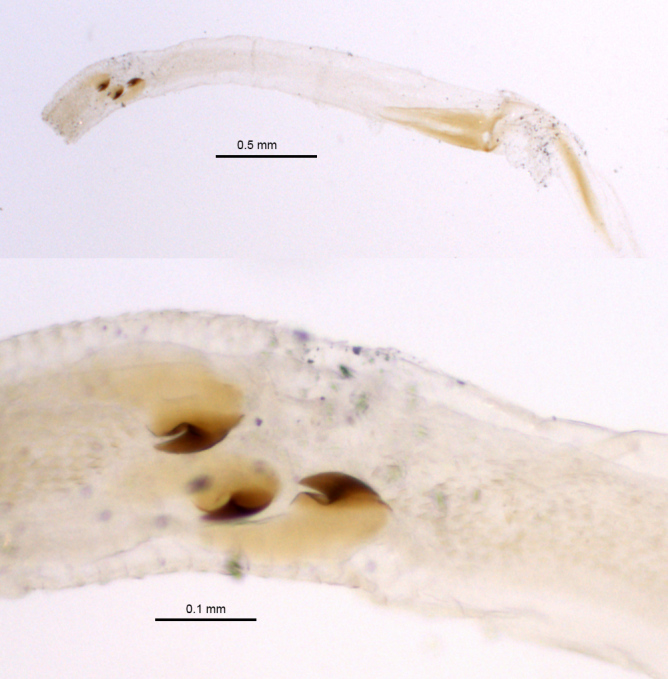
*Madecorphnus niger* Frolov, endophallus armature.

Type specimens of *Madecorphnus niger* were damaged (lacked endophalluses) and the species was described chiefly on the basis of the distinctive shape of the aedeagus. Examination of the newly available material showed that the endophallus armature is also quite distinctive. The armature consists of three similar, highly sclerotized, tooth-like sclerites (Fig. 1). Other sclerites or fields of spinules are absent.

### Madecorphnus falcatus

Paulian, 1992

#### Materials

- **Type status:** Other material. **Occurrence:** recordedBy: California Acad. of Sciences ant team; individualCount: 1; sex: male; **Taxon:** scientificName: Madecorphnus falcatus; genus: Madecorphnus; specificEpithet: falcatus; scientificNameAuthorship: Paulian, 1992; **Location:** country: Madagascar; verbatimLocality: Toamasina, Parcelle K9 Tampolo; verbatimElevation: 10 m; verbatimLatitude: 17°10'30''S; verbatimLongitude: 49°16'05''E; **Identification:** identifiedBy: Andrey V. Frolov; **Event:** eventDate: 19 April 2004; **Record Level:** collectionID: urn:lsid:biocol.org:col:35143; institutionCode: CAS; collectionCode: CASENT

#### Distribution

**Figure 2.**
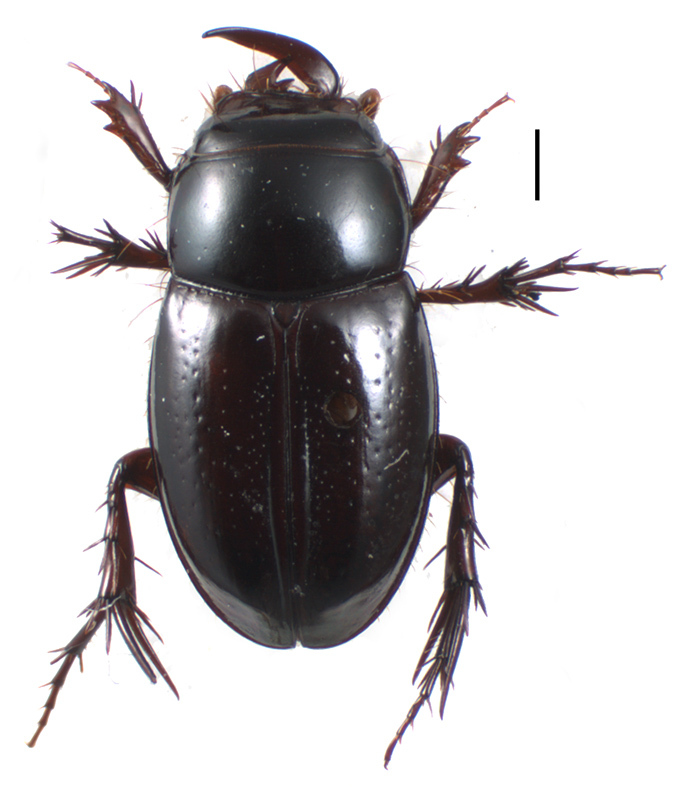
*Madecorphnus falcatus* Paulian, male, habitus.

**Figure 3.**
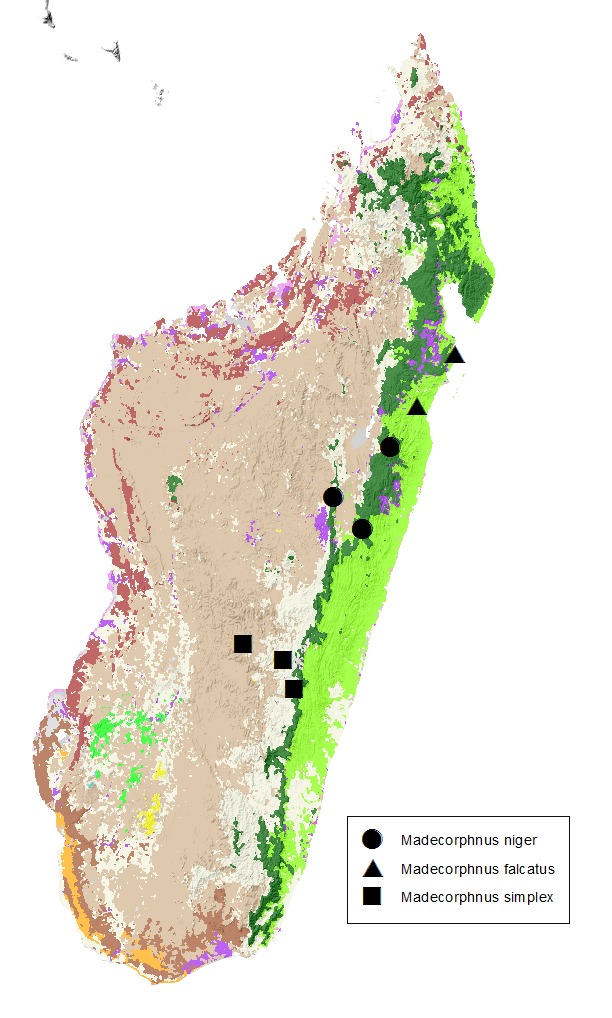
*Madecorphnus* spp. Locality map.

This species was known from the only type specimen collected in Antanambe, on the eastern coast of Madagascar. The additional specimen (Fig. 2) was collected some 100 km south (Fig. 3), in littoral forest litter. This species may be a littoral forest specialist.

### Madecorphnus simplex

Frolov, 2010

#### Materials

- **Type status:** Other material. **Occurrence:** recordedBy: R. Harin'Hala; individualCount: 1; sex: male; **Taxon:** scientificName: Madecorphnus simplex; genus: Madecorphnus; specificEpithet: simplex; scientificNameAuthorship: Frolov, 2010; **Location:** country: Madagascar; stateProvince: Fianarantsoa; verbatimLocality: Parc National Ranomafana, at broken bridge; verbatimElevation: 1110 m; verbatimLatitude: 17°10'30''S; verbatimLongitude: 49°16'05''E; **Identification:** identifiedBy: Andrey V. Frolov; **Event:** eventDate: 19-26 March 2002; **Record Level:** collectionID: urn:lsid:biocol.org:col:35143; institutionCode: CAS; collectionCode: CASENT
- **Type status:** Other material. **Occurrence:** recordedBy: Fisher, Griswold et al.; individualCount: 1; sex: male; **Taxon:** scientificName: Madecorphnus simplex; genus: Madecorphnus; specificEpithet: simplex; scientificNameAuthorship: Frolov, 2010; **Location:** country: Madagascar; stateProvince: Fianarantsoa; verbatimLocality: Foret d'Atsirakambiaty, 7.6 km 285° WNW Itremo; verbatimElevation: 1550 m; verbatimLatitude: 20°35'36''S; verbatimLongitude: 046°33'48''E; **Identification:** identifiedBy: Andrey V. Frolov; **Event:** eventDate: 22-26 January 2003; **Record Level:** collectionID: urn:lsid:biocol.org:col:35143; institutionCode: CAS; collectionCode: CASENT

#### Distribution

The species was described from one specimen from Ambatofitorahana. The new locality records include Itremo Massif and Ranomafana National Park (Fig. 3).

### Triodontus itremoi

Paulian, 1977

#### Materials

- **Type status:** Other material. **Occurrence:** recordedBy: Fisher, Griswold et al.; individualCount: 6 males and 10 females; **Taxon:** scientificName: Triodontus itremoi; genus: Triodontus; specificEpithet: itremoi; scientificNameAuthorship: Paulian, 1977; **Location:** country: Madagascar; verbatimLocality: Foret d'Atsirakambiaty, 7.6 km 285ТА WNW Itremo; verbatimElevation: 1550 m; verbatimLatitude: 20°35'36''S; verbatimLongitude: 46°33'48''E; **Identification:** identifiedBy: Andrey V. Frolov; **Event:** eventDate: 22-26 January 2003; **Record Level:** collectionID: urn:lsid:biocol.org:col:35143; institutionCode: CAS; collectionCode: CASENT
- **Type status:** Other material. **Occurrence:** recordedBy: B.L.Fisher et al.; individualCount: 4 males and 2 females; **Taxon:** scientificName: Triodontus itremoi; genus: Triodontus; specificEpithet: itremoi; scientificNameAuthorship: Paulian, 1977; **Location:** country: Madagascar; verbatimLocality: Toamasina Ambatovy, 12.4 km NE Moramanga; verbatimElevation: 1040 m; verbatimLatitude: 18°51'29" S; verbatimLongitude: 048°17'06" E; **Identification:** identifiedBy: Andrey V. Frolov; **Event:** eventDate: 5-8 March 2007; **Record Level:** collectionID: urn:lsid:biocol.org:col:35143; institutionCode: CAS; collectionCode: CASENT
- **Type status:** Other material. **Occurrence:** recordedBy: B.L.Fisher et al.; individualCount: 1 male; **Taxon:** scientificName: Triodontus itremoi; genus: Triodontus; specificEpithet: itremoi; scientificNameAuthorship: Paulian, 1977; **Location:** country: Madagascar; verbatimLocality: Toamasina Ambatovy, 12.4 km NE Moramanga; verbatimLatitude: 18°50'22" S; verbatimLongitude: 048°18'30" E; **Identification:** identifiedBy: Andrey V. Frolov; **Event:** eventDate: 4-7 March 2007; **Record Level:** collectionID: urn:lsid:biocol.org:col:35143; institutionCode: CAS; collectionCode: CASENT

#### Distribution

**Figure 4.**
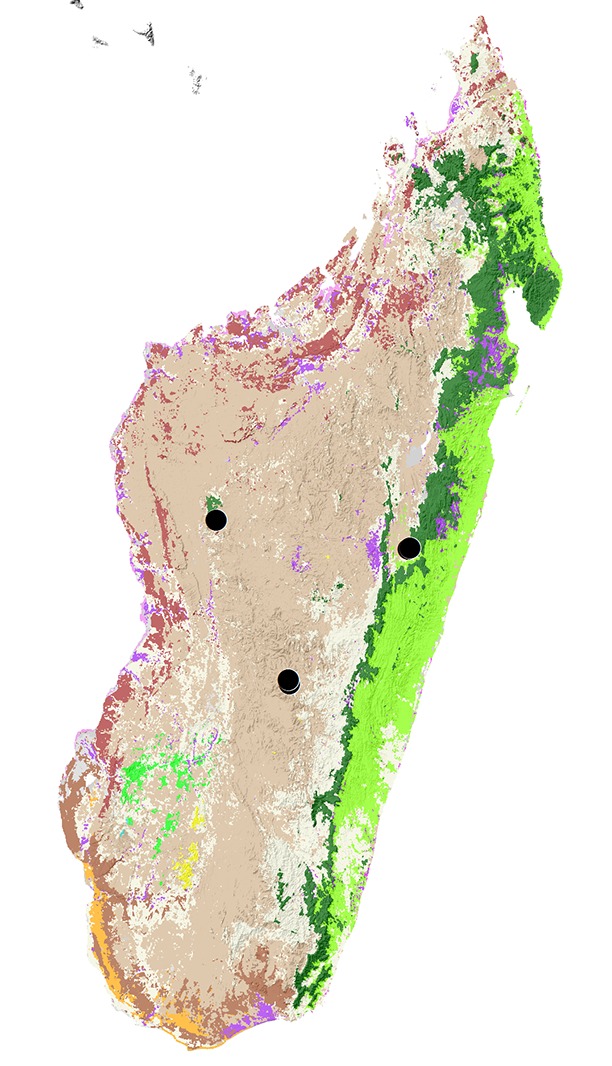
*Triodontus itremoi*. Locality map.

This species was described from two rather distant localities, in the Itremo Massif and Tsiroanomandidy (Bongolava district). New records extend the known species range to the eastern slopes of the central plateau occupied by the largest remnants of rain forest (Fig. 4).

## Supplementary Material

XML Treatment for Madecorphnus niger

XML Treatment for Madecorphnus falcatus

XML Treatment for Madecorphnus simplex

XML Treatment for Triodontus itremoi
